# Multiscale imaging on *Saxifraga paniculata* provides new insights into yttrium uptake by plants

**DOI:** 10.1038/s41598-022-23107-x

**Published:** 2022-10-30

**Authors:** Till Fehlauer, Blanche Collin, Bernard Angeletti, Mohammad Mustafa Negahi, Cédric Dentant, Perrine Chaurand, Claire Lallemand, Clement Levard, Jérôme Rose

**Affiliations:** 1 Aix Marseille Univ., CNRS, IRD, INRAE, Coll. France, CEREGE, Aix-en-Provence, France; 2 Parc national des Écrins, Domaine de Charance, 05000 Gap, France; 3 Univ. Grenoble Alpes, CNRS, Sciences Po Grenoble, Pacte, 38000 Grenoble, France

**Keywords:** Biogeochemistry, Ecology, Plant physiology, Pollution remediation, Environmental impact

## Abstract

Yttrium (Y) has gained importance in high tech applications and, together with the other rare earth elements (REEs), is also considered to be an emerging environmental pollutant. The alpine plant *Saxifraga paniculata* was previously shown to display high metal tolerance and an intriguing REE accumulation potential. In this study, we analysed soil grown commercial and wild specimens of *Saxifraga paniculata* to assess Y accumulation and shed light on the uptake pathway. Laser ablation inductively coupled plasma mass spectrometry and synchrotron-based micro X-ray fluorescence spectroscopy was used to localise Y within the plant tissues and identify colocalized elements. Y was distributed similarly in commercial and wild specimens. Within the roots, Y was mostly located in the epidermis region. Translocation was low, but wild individuals accumulated significantly more Y than commercial ones. In plants of both origins, we observed consistent colocalization of Al, Fe, Y and Ce in all plant parts except for the hydathodes. This indicates a shared pathway during translocation and could explained by the formation of a stable organic complex with citrate, for example. Our study provides important insights into the uptake pathway of Y in *S. paniculata*, which can be generalised to other plants.

## Introduction

Yttrium (Y) is a trivalent transition element and is considered as a rare earth element (REE). Today, REEs are critical resources in many countries as they are essential for high-tech applications but economically attractive deposits are rare^1,2^. However, concerns are being raised not only because of geopolitical issues, but also because of the environmentally damaging extraction methods used and the lack of knowledge on their environmental fate^3,4^. Scientific findings remain elusive, especially regarding their effects on plant growth, as both positive and negative effects have been observed^5,6^.

Plants can serve as local indicators of environmental health and are a major entryway for contaminants into the food chain^7^. A better knowledge of the effects on plant metabolism and the uptake mechanisms is therefore essential to evaluate the environmental risks linked to these emerging pollutants.

So far, most studies of the effect of REEs on plants, have focussed on Ce, La, Eu or Nd^3,8^. It has been discovered that they affect Ca dependent processes in the plant metabolism due to their similar ionic radii^9–11^. The proximity to physiological functions of Ca is usually attributed to all REEs, but recent studies point to differences in the behaviour of light REEs (LREEs, from La to Eu) and heavy REEs (HREEs, from Gd to Lu + Y)^12–14^. According to these studies, shared uptake pathway with Ca only applies to LREEs, while the HREEs uptake pathway is still the subject of many questions^3^. Yuan et al. suggested a shared uptake pathway between HREEs and Al after exposing the REE hyperaccumulating plant *Phytolacca americana* to a mixture of heavy and light REEs^15^. However, this hypothesis is based on a hydroponic culture and requires further substantiation. Even though hydroponic cultures are very useful to observe the effects of a specific treatment under controlled conditions and enable preliminary hypotheses, they do not account for the complexity of the interactions with the rhizosphere that take place in soil^5^.

Furthermore, current studies on REEs in plants focus on a small number of species, particularly on REE accumulating plants like *Phytolacca americana* and *Dicranopteris linearis*^13,16–20^. Accumulating plants are an excellent way to study elemental uptake pathways, but it is important to diversify the number of plant species studied, since elemental uptake pathways are species dependent and may even vary from one population of the same species to another^21^.

To improve our understanding of REE behaviour in plants, we chose to focus on Y as a proxy for HREEs, and to use *Saxifraga paniculata* (*S. paniculata*), a rugged alpine plant species shown to have REE accumulation potential^22,23^. The REE affinity of *S. paniculata* was discovered in a previous study that examined elemental uptake patterns in several alpine plant species^23^. *S. paniculata* showed a high bioconcentration factor (1.7 ± 0.8) for Y despite the fact that the total soil concentrations were low (4.22 ± 1.04 mg/kg)^23^. In the present study, we evaluate the phytoextraction potential of *S. paniculata* after cultivation in a Y doped soil with the aim of shedding light on the uptake pathway of Y and of identifying possible interactions between Y and other chemical elements. Our approach combined the high spatial resolution of synchrotron-based micro X-ray fluorescence (µXRF) with the high sensitivity of laser ablation inductively coupled plasma mass spectrometry (LA-ICP-MS). This approach enabled us to pinpoint the exact location of Y in the different plant tissues and to detect very low elemental concentrations in order to explore colocalization with other chemical elements. Localization is an important step in understanding elemental uptake pathways in plants and high spatial resolution mapping of REEs almost never been attempted in plant tissues^4,9,24,25^.

## Materials and methods

### Plant growth

Individuals from two different populations of *S. paniculata*, were transplanted to a Y-doped soil. Wild plants, were collected at Jas Roux, an undisturbed and naturally metal-rich site in the French Alps^23,26^. The collection of the wild specimens was performed with the permission of the Ecrins National Parc and in accordance with relevant guidelines and regulations. The soil parameters of Jas Roux are presented and discussed in our previous article^23^. Commercial plants from a plant nursery (staudenonline.de) have been selected as a second population to investigate the possibility of locally modified uptake mechanisms in *S. paniculata* from Jas Roux.

Before transplantation, the former growth-substrate was removed with tap water, and the roots were cleaned with deionised water. A total of three commercial plants were grown on Y-doped soil and two commercial plants were grown on non-doped soil as controls. Six wild plants sampled at the Jas Roux site were grown on Y-doped soil, and four individuals were grown on non-doped soil.

A standardised soil obtained from the Speyer agricultural and forestry research institute in Germany was used for this experiment (SM Table 1). The soil is a non-fertilised clayey loam taken from a meadow with apple trees. The initial Y concentration measured in the untreated soil was 5 mg/kg which is in the lower range of the usual topsoil concentrations (7–60 mg/kg)^6,11,27–29^. The soil was doped with Y to ensure sufficient Y would be detected within plant tissues using imaging techniques. To increase the total concentration of Y in the soil, 1.66 g of Y(NO_3_)_3_ (Yttrium (III) nitrate hydrate—REacton^®^, 99.99%), dissolved in 150 mL ultrapure water (18.2 MΩ⋅cm) were added per kg of dry soil. An incubation period of one month was respected before each individual plant was transplanted into 250 g of soil respectively. During the incubation period, the water content of the soil was maintained at about 70% of the field capacity.

The two populations of *S. paniculata* were not cultivated simultaneously but were divided into two separate experiments, due to a lack of space in the growth chamber. The soil for each experiment was prepared independently in large quantities and in each case, only a portion was used. The total Y concentrations in the doped soil were 373 ± 17.3 mg/kg DW (n = 3) for the commercial plants and 216 ± 21.8 mg/kg DW (n = 6) for the wild plants (SM Table 3). The difference between the two soil concentrations of Y was probably caused by a heterogeneity of Y distribution in the soil. However, within the same population, the concentrations are homogeneous, as can be seen from the low standard deviations.

The mobile fraction of Y in the doped soil was estimated using CaCl_2_ extraction (0.001 mol/L) at a liquid to solid ratio of 10 L/kg dry matter^30^. Y mobility was very low (0.1 µmol/L) and did not exceed 0.05% of the total concentration.

*Saxifraga paniculata* plants were grown for a period of four months under controlled conditions in a growth chamber (Aralab^®^, FitoClima 1200) and watered with deionised water twice a day (SM Fig. 1). The plants showed no obvious signs of toxicity induced by the Y treatment. At harvest, there was no significant difference in fresh plant weight between the Y-treated samples and the controls.

### Sample preparation

After 4 months of growth, the plants were harvested and prepared for the different analyses. For the determination of total elemental concentrations, the soil was carefully removed from the roots. The plants were then separated into leaves, stem and roots. All plant parts were carefully washed with ultrapure water (18.2 MΩ⋅cm) and dried at 40 °C for 48 h. Subsequently they were ground into a fine powder in a Retsch^®^ Mixer Mill MM 400 for 2 min at 20 Hz. Soil samples were dried at 40 °C for 72 h and ground for 2 min in a planetary mill at 400 rpm to destroy soil aggregates (Fritsch^®^, Pulverisette 6). Inductively coupled plasma mass spectrometry (ICP-MS, PerkinElmer^®^ NexIon 300X) analysis was performed on three Y-treated and two untreated commercial plants and six Y-treated and four untreated wild plants.

Roots destined for LA-ICP-MS and µXRF analysis were gently isolated from the root ball without removing the adhering soil. They were directly embedded in tissue-tec^®^ OCT compound using 6 × 12 × 5 mm moulding cups, immersed in liquid nitrogen and stored at − 80 °C until analysis. The stems were also embedded in tissue-tec^®^ OCT compound and immersed in liquid nitrogen. The leaves were separated individually from the living plants and directly immersed in liquid nitrogen before being freeze-dried.

The frozen, OCT embedded samples (stems and roots) were then cut into fine 100 µm cross sections by cryostat (Leica^®^ CM1900). The cross sections were fixed between two layers of Kapton^®^-film and freeze-dried for 24 h (Cryotec^®^ laboratory freeze dryer Crios). The best-preserved cross sections were identified under an optical microscope and analysed by µXRF and LA-ICP-MS. Side roots of at least 0.5 mm in diameter have been sampled for imaging analysis. Root tips were excluded from the analysis due to their different structure and functionalities. Care was taken to ensure that the cross-sections selected for µXRF and LA-ICP-MS analysis were two consecutive root sections for each treatment and population.

### Inductively coupled plasma mass spectrometry: elemental analysis

100 mg of each sample were digested in acid using a microwave MLS UltraWave Thermo-Scientific^®^. Mineralisation was performed using 4 mL of HNO_3_ and 2 mL of H_2_O_2_ for the plant samples and 3 mL of HNO_3_, 3 mL of HCl and 0.5 mL of HF for the soil samples.

The ICP-MS calibration curves were established using multi-solution CCS4, CCS5 and CCS6 standards purchased from Inorganic Ventures^®^ (New Jersey USA) diluted in 2% HNO_3_ solution. The internal standard was ^103^Rh. Oak leaves (V464) provided by the French National Institute for Agriculture Food and Environment (INRAE) were used as the external reference for plant samples. EnviroMAT drinking water EP-L-3 and subterranean water ES-H-2 (SCP Sciences) were used as internal references and for quality control. The recovery rate and coefficients of variances of all certified elements of the external and internal references are listed in SM Table 2. The detection limit for each element was determined using handling blanks analysed at each ICP analysis (SM Table 2). Statistical differences in total elemental concentrations were tested using a pairwise Mann–Whitney U test with the “stats” package in the open-source software Rstudio^31,32^.

The translocation factor (TF) was calculated to identify *S. paniculata*’s uptake strategy. The TF is obtained by dividing the elemental concentrations in the aerial parts by the concentrations in the roots^33^.

### Synchrotron-based micro X-ray fluorescence: high spatial resolution

Scanning X-ray fluorescence microscopy analysis was performed using the Nanoscopium beamline of synchrotron Soleil (Paris, France) with the ring running at 2.75 GeV and 500 mA. Nanoscopium X-ray energy can be tuned from 5 to 25 keV, which comprises the K absorption edges of the elements of interest in this study (e.g. Y = 17,038 eV)^34^. The monochromatic X-ray beam was focused on the sample by a Kirckpatrick–Baez nano-focusing mirror^35^. The samples were mounted on a 3-axis sample positioning stage equipped with a stepper motor for coarse pitch and a piezo-motor for fine pitch. With this setup, Y-concentrations down to 10 mg/kg can be reliably detected. Freeze-dried leaf, stem and root cross sections of commercial and wild *S. paniculata* were analysed using fast continuous scanning (FLYSCAN). This technique makes it possible to create mm^2^ sized elemental maps with a resolution down to 0.4 µm/pixel^36^. For each pixel, an XRF spectrum was measured by two identical silicon drift detectors of 50 mm^2^ active surfaces (VITUS H50, KETEK GmbH) coupled to XMAP (XIA LLC) fast digital multichannel analyser cards^37^. X-ray light intensity and exposure time were adjusted for each sample to avoid saturation of the two fluorescence detectors.

During data processing, the signal of the two detectors was summed up and the deadtime was corrected using the open-source software Fiji^38^. The spectra were saved as 16 bit tiff stacks. The elemental maps were created using the ROI imaging tool of open-source software pyMCA, which makes it possible to analyse XRF datasets and identify the elemental peaks^39^. For each XRF spectrum, we selected the elements that enabled the best fit. Once the spectra were fitted, the background was removed using “Subtract SNIP 1D Background”. The elemental maps were extracted by choosing the corresponding energy range on the spectrum (SM Fig. 3). The resulting elemental maps were edited and analysed using the open-source software Fiji^38^.

### Laser ablation coupled with ICP-MS: high sensitivity and localization of light elements

LA-ICP-MS analysis was used as a complementary analysis to the µXRF analysis. The technique allows to localize light elements such as Al and P that were impossible measure with µXRF. Moreover, the high sensitivity of LA-ICP-MS enabled us to detect lower concentrations even if the spatial resolution (1 px = 10 µm) was considerably reduced compared to µXRF (1 px = 0.4 µm).

For LA-ICP-MS analysis, the LSX-213 Laser Ablation System by CETAC Technologies^®^ (Nd-YAG laser, wavelength 213 nm) coupled with ICP-MS (NexiION 300 × ICP-MS, PerkinElmer) were used. The parameters used for the scans were a frequency of 10 Hz, a laser beam diameter of 10 μm and a laser scan speed of 10 μm/s (laser power density: 3.74 GW/cm^2^, fluence: 18.72 J/cm^2^). With this technique, the detection limit for Y was 1 mg/kg. Due to the high frequency only a limited number of elements could be analysed by ICP-MS. We selected ^24^Mg, ^27^Al, ^31^P, ^39^K, ^43^Ca, ^57^Fe, ^63^Cu, ^66^Zn, ^89^Y and ^140^Ce. Ce was chosen as the second REE because it is the most naturally abundant REE and belongs to the group of LREEs^6^. The respective elemental maps were created using the open-source software Fiji^38^. Root cross sections, stem cross sections and leaves of plants of both origins were analysed by LA-ICP-MS, and elemental colocalizations were identified using principal component analysis (PCA) and pairwise correlation analysis. Each point of measurement (pixel) was considered as an individual (row) and each element as a variable (column). Positively correlated elements in the resulting PCA show high values for the same pixels of the respective elemental map, indicating a colocalization of these elements. One example of such a colocalization can be seen in SM Fig. 7, which shows a strong signal for different elements in the same pixels. The PCAs were performed on regions of interest (ROIs) drawn on the unscaled elemental maps (SM Fig. 6).

Outliers were identified and removed using density-based spatial clustering of applications with noise (dbscan)^40^. Spearman correlations were chosen for correlation analysis as no linear relationship was found between elements. All analyses were performed using the open-source software Rstudio and the packages “fpc”, “factoextra”, “FactoMineR”, “psych” and “corrplot”^32,41–45^. We encountered an ICP acquisition problem in the elemental maps of P in all samples of the commercial plants and in the elemental map of Fe in the leaves of the wild plants which is why they were excluded from their respective PCAs (Fig. 5).

## Results and discussion

### Y uptake by two different populations of *S. paniculata*

**Figure 1:**
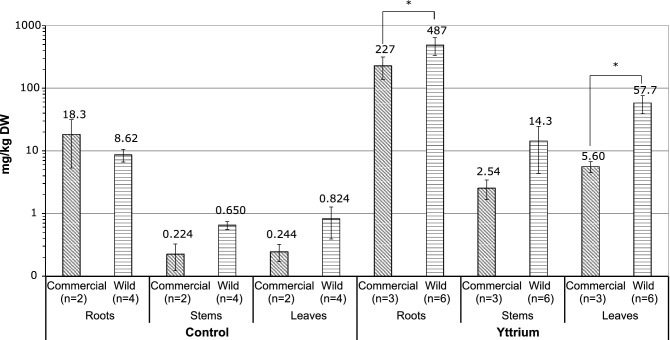
Total concentrations of Y in the different plant parts (Roots, Stem, Leaves) of *Saxifraga paniculata* grouped according to origin (Commercial, Wild) and treatment (Control, Yttrium); Significant differences (p < 0.05) between the two origins according to a Wilcoxon rank-sum test are indicated by asterisks.

The total concentrations of Y measured in the roots, stems and leaves of commercial and wild *S. paniculata* are shown in Fig. 1. In the roots, the concentration of Y was 487 ± 153 mg/kg in the wild plants and 227 ± 88.6 mg/kg in the commercial plants. The concentrations in the stems were lower at 14.3 ± 9.97 mg/kg and 2.54 ± 0.889 mg/k, respectively. The concentration of Y in the leaves was 57.7 ± 18.4 mg/kg in the wild plants and 5.60 ± 1.11 mg/kg in the commercial plants. The wild plants had significantly higher concentrations in the roots and the leaves than the commercial plants. The fact that these differences exist despite of the initially lower Y concentrations in the doped soil of the wild plants (see “Plant growth” section), suggests an increased uptake potential for this population. The high concentrations in the roots compared to those in the aerial parts are in agreement with current knowledge according to which Y and other REEs are preferentially stored in the roots^6,28^. However, this may include an adsorbed and an absorbed fraction.

To evaluate the accumulation potential of a given plant species, it is essential to compare it with other species grown in similar experimental conditions. The only comparable study that quantified Y in plants growing on a doped soil was performed by Purwadi et al.^17^. These authors exposed two potentially REE hyperaccumulating plants to a soil doped with 1000 mg/kg of Y, La and Nd and concentrations of 45 ± 40 mg/kg Y were measured after a 6-months growing period in old leaves of *Melastoma malabathricum* and 250 ± 220 mg/kg Y in old fronds of *Dicranopteris linearis*. Although these concentrations are considered high compared to typical REE concentrations in plants, they are well below the threshold of 1000 mg/kg set for REE hyperaccumulation. The authors thus concluded that the hyperaccumulation status of the two species cannot conclusively be confirmed^17^. Consequently, this also applies to the Y accumulation capacity of *S. paniculata* in our study. The low translocation factors (TFs) of 0.03 ± 0.01 of the commercial plants and 0.14 ± 0.08 of the wild ones did not indicate any Y hyperaccumulation trait in *S. paniculata* either. Nevertheless, it should be noted that REE extraction potential is usually evaluated by summing all extracted REEs, and further evaluation of *S. paniculata* may thus be worthwhile^20,46^.

A correlation analysis of the total elemental concentrations in the leaves is often performed as a first approach to identifying competitive or synergetic effects between different chemical elements and to establish a preliminary hypothesis concerning elemental translocation^13,47^.

However, it should be kept in mind that this approach does not consider the spatial distribution of the elements within plant tissues, but only shows the elemental correlations among samples.

**Figure 2:**
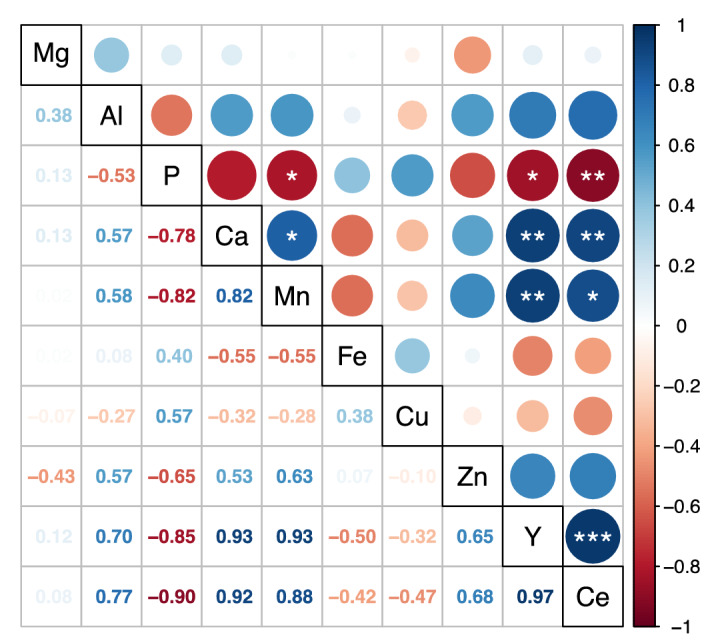
Correlation between total concentrations of different chemical elements in the leaves of *Saxifraga paniculata* of both origins (n = 9) illustrated by a correlation plot of Spearman’s rank coefficients with significant values indicated by asterisks (significance levels = 0.5, 0.01, 0.001; p.adjust = Benjamini and Hochberg).

Commercial and wild plants are represented together on the same correlation plot since elemental correlations were similar (Fig. 2). The graphic only shows leaf concentration correlations, where elemental sorting is most visible, whereas correlations in the roots have little informative value (SM Fig. 2). Furthermore, total concentrations in the roots are often biased by adsorbed elements or incorporated soil particles, and do not reflect the uptake fraction very well^48^.

Figure 2 shows that Y is in significantly positively correlated with Ca, Mn, Ni, Ba and Ce but in negatively correlated with P. A negative correlation between P and REEs has already been observed in hydroponically grown maize plants and in *Arabidopsis thaliana*^2,49^. Saatz et al. suspect that this is due to a reprecipitation of Y phosphates inside the plant root once the REEs are absorbed^25^. The positive correlation between the cations Ca, Mn, Ni, Y, Ba and Ce are in accordance with the findings of our previous study and validate the hypothesis of an affinity for divalent cations and REEs in *S. paniculata*^23^. One hypothesis that could explain these affinities is increased activity or overexpression of one or more transporters with low elemental selectivity of the NRAMP or the ZIP family, for example^50–52^. Another hypothesis is increased cation mobilisation due to rhizosphere acidification or increased production of mobilising root exudates, for example. In this regard, we suspect a link with P acquisition since REEs often occur as insoluble phosphates such as xenotime (HREEs) and monazite (LREEs) in soils^53^. Phosphate mobilising root exudates comprise protons, phosphatases and carboxylates^54^. It has been reported that carboxylates for instance, not only mobilise P, but also transition metal cations such as Mn, Fe, Cu and Zn^55,56^. The intensity of carboxylate production, however, depends on soil properties, plant species and phosphate supply^57^.

To further understand Y uptake, it is necessary to localise Y in the tissues, since a study of the total concentrations only provides limited insights. To this end, µXRF and LA-ICP-MS were conducted on root cross sections to localise Y within the roots of *S. paniculata* and to compare the spatial distribution with other elemental maps to identify potential colocalizations.

### Y uptake in *S. paniculata*: high resolution elemental mapping of roots

**Figure 3:**
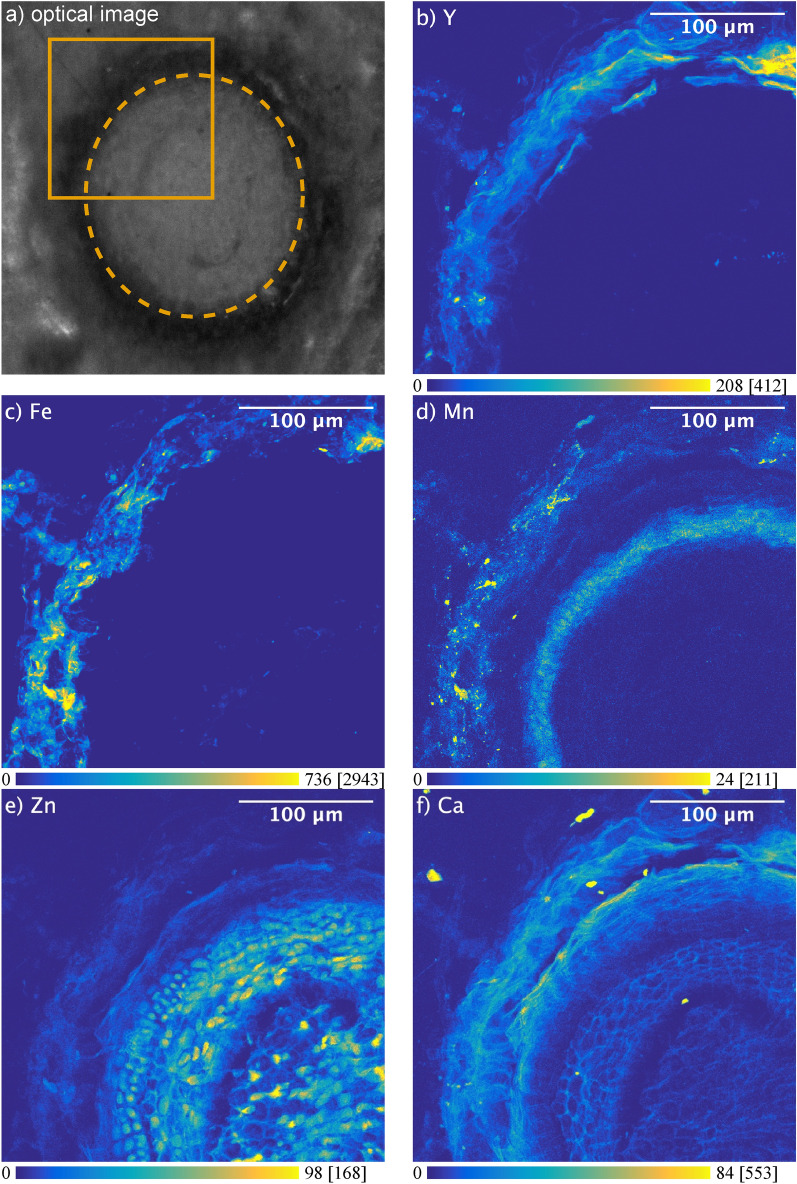
µXRF elemental maps (arbitrary unit) showing the distribution of Y, Fe, Mn, Zn and Ca in a root cross section of wild *Saxifraga paniculata* grown in Y doped soil; (**a**) shows the scanned region and the outline of the root; (**b–f**) show elemental maps of K-emission lines; X-ray spot-size: 0.4 µm; Counting-time: 40 ms/px; Gap-size: 100 µm × 60 µm; Contrast: 0.3% saturated pixels; Maximum pixel values in brackets.

**Figure 4:**
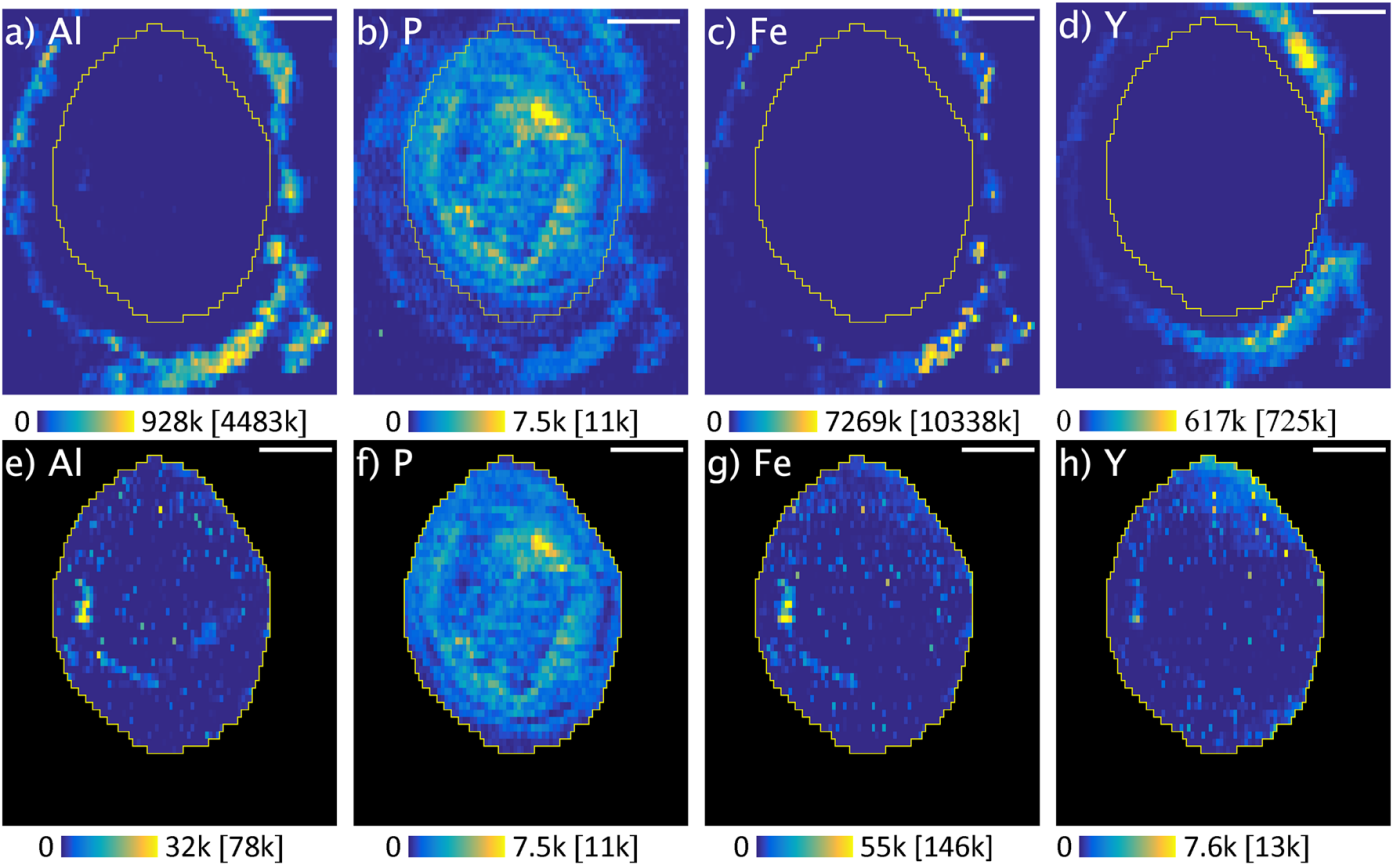
Scaled LA-ICP-MS elemental maps (arbitrary unit) for Al, P, Fe and Y of a root cross section of wild *Saxifraga paniculata*; Maps (**a–d**) show the complete images, while maps (**e–h**) only show a region of interest inside the root; Contrast: 0.3% of saturated pixels; Maximum pixel values are indicated in brackets; Scale bar: 100 µm; Map-size: 463 × 53 0 µm; Spot-size: 10 µm; Frequency: 10 Hz; Scan-speed: 10 μm/s; Laser power density: 3.74 GW/cm^2^; Fluence: 18.72 J/cm^2^.

The spatial distribution of chemical elements in root cross sections of wild *S. paniculata* is shown in Figs. 3 and 4. Two complementary imaging techniques were used to map Y, Fe, Mn, Zn, Ca (Fig. 3) and Al, P, Fe and Y (Fig. 4) on consecutive root cross sections, at different spatial resolution using synchrotron based µXRF and LA-ICPMS.

The maps reveal very different distributions depending on the chemical element. While Fe and Y appear to remain mainly in the epidermis region, the distribution of Zn, Ca or Mn differs greatly and each of these elements is accumulated in a different root tissue (Fig. 3d–f). Mn remains in the cortex and is accumulated around the stele. This might be the result of Mn being largely blocked by the Casparian strip and is consistent with observations that have been made on barley roots using LA-ICP-MS^58^. On the other hand, Zn and Ca are distributed over the entire cross section (Fig. 3e,f). Zn appears to be absorbed without restriction and even accumulated in the root cells, while Ca surrounds the cells and largely retained in the epidermis region. These observations confirm current knowledge on the uptake pathways of Ca and Zn. While Ca is usually very abundant in the cell membranes and absorbed via the apoplastic pathway, Zn is absorbed via the symplastic pathway^59^. These elemental maps allow to clearly identify individual cells and provide an insight into the root structure. A comparison with the elemental map of Y shows clear differences in the distribution (Fig. 3b). Figures 3b and 4a reveal that the high concentrations of Y measured in the roots of wild *S. paniculata* via ICP-MS are mainly localised in the epidermis region of the root and barely penetrate the interior. The same phenomenon was observed on the root samples of commercial *S. paniculata* (SM Fig. 4).

This observation weakens the common assumption of REEs mainly being blocked by the Casparian strip^60^. Another theory is the binding to carboxylic groups in the cell walls of the apoplast with which polyvalent cations like REEs strongly interact^25,48^. However, there are no cellular structures on the elemental map of Y such as those visible on the elemental map of Ca, that could underpin this hypothesis (Fig. 3b,f).

Y might also be retained outside the root tissue by mucilage for example. Mucilage is a gelatinous material consisting of high molecular weight polysaccharides surrounding the root^61^. Mucilage is known to have a detoxifying effect on Al and might also block other trivalent cations like Y^59^. This hypothesis is supported by the fact that Y, Al and Fe show very similar elemental maps (Figs. 3b,c, 4a,c,d).

Saatz et al. who examined the Y distribution in the roots of hydroponically grown maize plants using LA-ICP-MS made a similar observation^25^. These authors hypothesised that the REEs are accumulated in iron oxy-hydroxide coatings on the surface of the root^2^. However, such iron oxy-hydroxide coatings, also known as iron root plaque, are usually linked to wetland plants and have not been documented on plants grown under permanent oxic conditions^62,63^.

The selection of a region of interest (ROI) covering only the interior of the root and readjustment of the contrast revealed elemental localizations inside the root (Fig. 4d–f). Y and Al distributions seem to correlate not only outside the root but also inside, as they share hotspots (Fig. 4d,f). Reprecipitation of Y-phosphate inside the root, as suspected by Saatz et al.^25^, is not supported by our data, as the distribution of P and Y is very different (Fig. 4d,e). Nevertheless, such a colocalization could be masked by overall high concentrations of P. Colocalizations between elements is an indication of a similar behaviour and could imply a shared uptake pathway. In the following section, we use a statistical approach to explore colocalizations between multiple elements all at once.

### Y translocation in *S. paniculata*: elemental colocalizations in different plant parts

**Figure 5:**
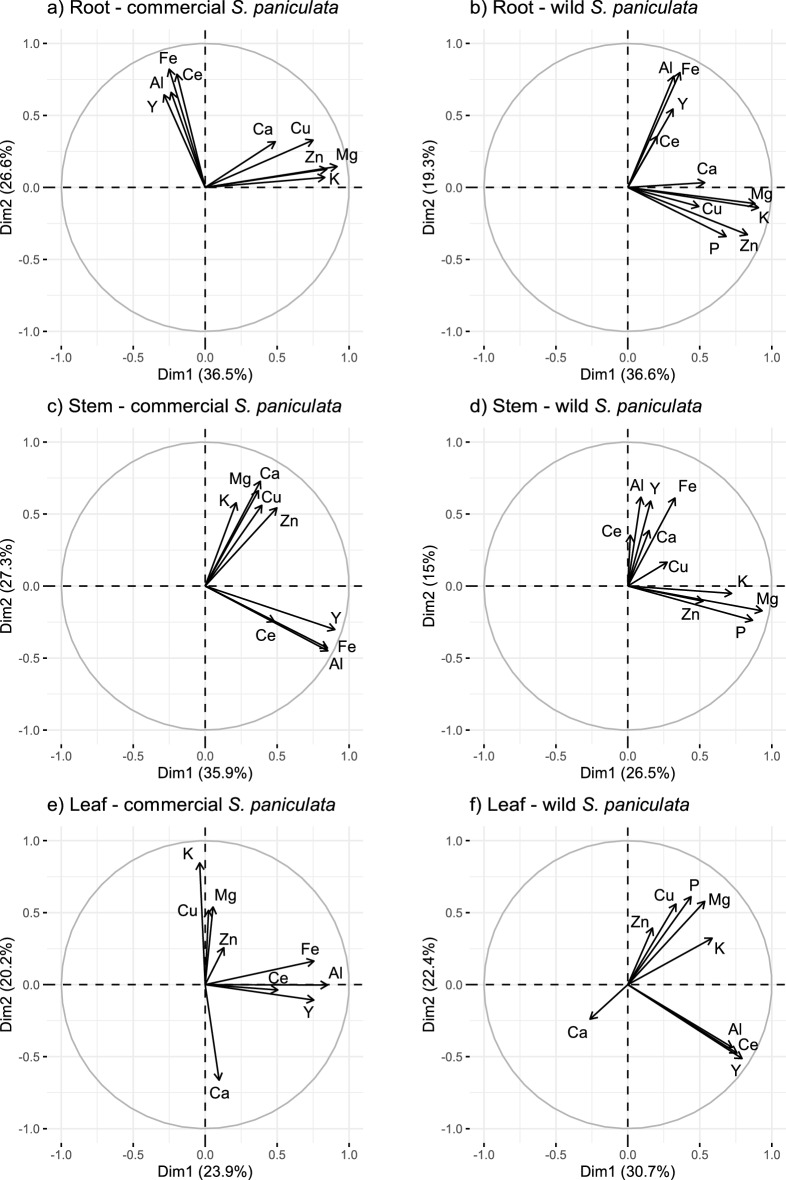
Elemental colocalizations represented by PCA loadings plots based on pixel intensities of elemental maps; Elemental maps were obtained by LA-ICP-MS analysis of cross sections of roots (**a,b**), stems (**c,d**) and leaf surfaces (**e,f**) of commercial (**a,c,e**) and wild (**b,d,f**) *Saxifraga paniculata*. ^24^Mg, ^27^Al, ^31^P, ^39^K, ^43^Ca, ^57^Fe, ^63^Cu, ^66^Zn, ^89^Y and ^140^Ce were selected for the analysis; P was excluded from (**a–c**) and Fe from (**f**) due to acquisition problems; The total number of pixels in each map are: (**a**) 778 [6]; (**b**) 1830 [1]; (**c**) 2717 [20]; (**d**) 4123 [0]; (**e**) 11,130 [41]; (**f**) 10,477 [11]; Number of removed outliers are in indicated in brackets.

The variable plots of the PCA of the elemental maps of the different plant parts are shown in Fig. 5. The respective Spearman coefficients are shown in SM Fig. 5 and the associated elemental maps of Y in SM Fig. 6. The results of this colocalization analysis are of a different nature than the correlations between total elemental concentrations shown in Fig. 2, as the colocalization analysis also considers the spatial distribution of elements.

The first two dimensions of the PCAs on the root cross section, the stem cross section, and the leaf surface of the commercial *S. paniculata* explained, respectively, 63.1%, 63.2% and 44.1% of the total variance, while the cumulative percentages for wild *S. paniculata* were 51.2%, 40.5% and 53.1%, respectively. All the PCAs were composed out of two independent groups of positively correlated elements. Al, Fe, Y and Ce formed a persistent group in roots, stems and leaves of plants of both origins (Fig. 5).

**Figure 6:**
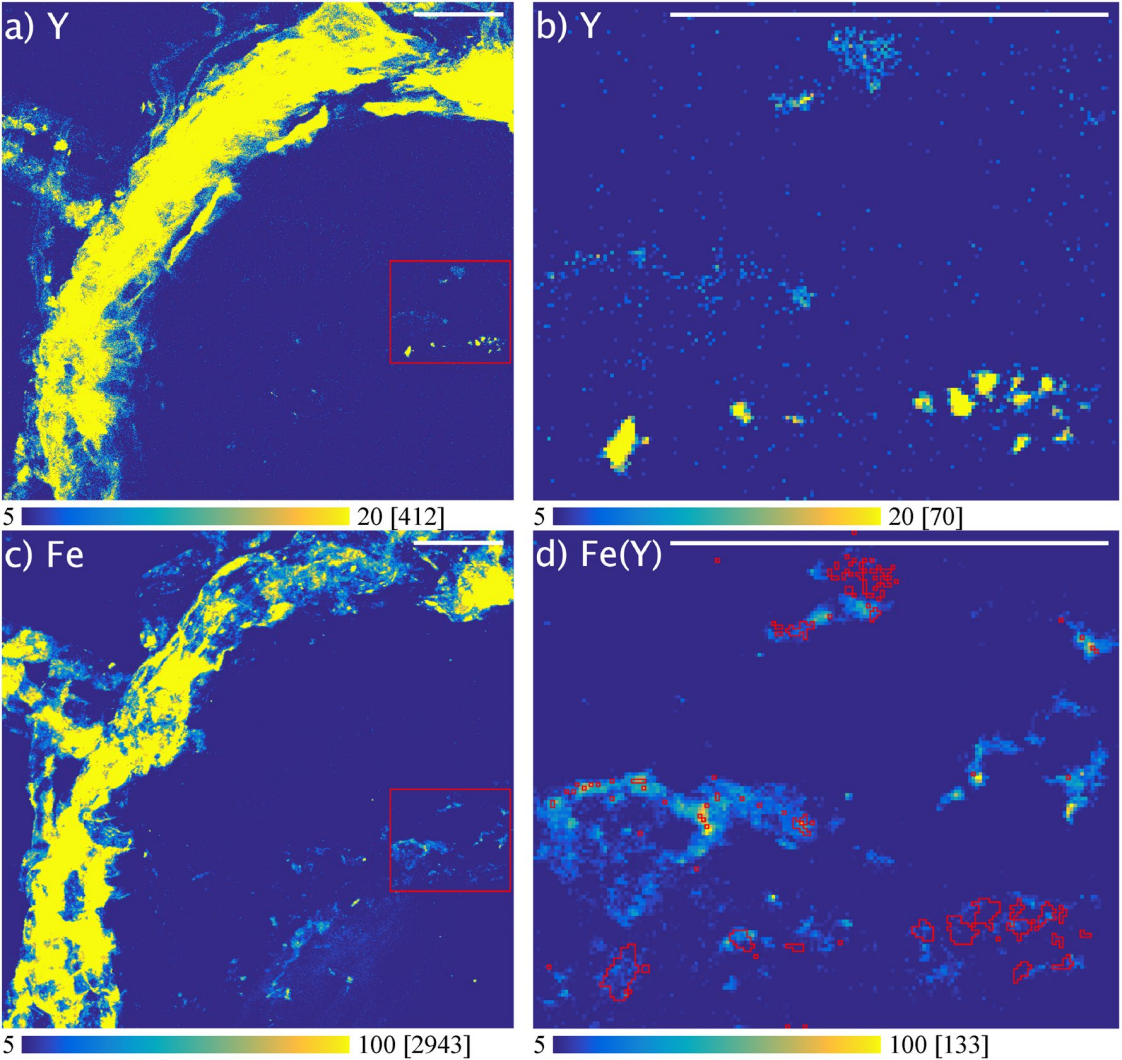
Colocalization of yttrium (Y) and iron (Fe) in region of interest (ROI) containing Y hotspots located in the cross section of wild *Saxifraga paniculata* roots analysed by µXRF; (**a,c**) show the elemental maps of Y and Fe with the outline of the ROI; (**b**) shows the elemental map of Y in the ROI containing Y hotspots; (**d**) Elemental map of Fe inside the ROI and outline of Y hotspots; Pixel-values ≥ 9 (arbitrary unit) were taken into account for outlining the Y hotspots; Colormap minima and maxima adjusted to increase contrast; Maximum pixel values are indicated in brackets; Scale bars: 50 µm.

The fact that Y (HREE) and Ce (LREE) remain colocalized in all plant parts suggests no difference in the handling of the two REEs. Unfortunately, it was not possible to further examine this colocalization in µXRF because of the low concentrations of Ce and the overlapping X-ray fluorescence emission lines. However, it was possible to examine the colocalization between Fe and Y (Fig. 6). Figure 6b shows small spots of Y located inside the roots. Superimposing these hotspots on the elemental map of Fe shows a colocalization between the two elements (Fig. 6d). Among all elements isolated from the XRF spectrum, Fe had the highest correlation coefficient with Y in this zone (Spearman’s rank correlation value = 0.193) (SM Table 4). The colocalization between Fe and Y measured with LA-ICPMS was therefore confirmed at the nanoscale resolution with µXRF.

Regarding the involved transporters and channels that allow Y to enter the root, only speculations are possible. It is usually assumed that REEs are absorbed through Ca-channels^64^. However, due to the similarities in the localization of Y and Al we rather support the hypothesis proposed by Yuan et al., according to which only LREEs are be absorbed through Ca channels, and HREEs are hypothesised to share a pathway with Al^15^. Our results are also partly in accordance with the observation made by Brioschi et al. in beech and oak. These authors found a strong correlation between the concentrations of REEs and the concentrations of Al, Fe, Mg, K and P in sap samples, and suggested a shared pathway between REE and macronutrients^60^. Our experimental observations using multiscale imaging in *S. paniculata*, also revealed shared mechanisms for REEs, Al, and Fe, but not for the macronutrients Mg, K and P.

It is plausible that Y enters the root via the same transporters as Al since an upregulation of Al-transport related proteins and transporters (NIPs, ALMT) has been reported in hydroponically grown *Phytolacca americana* under Y stress^65^. The persistent colocalization between Al, Fe, Y and Ce inside the plant suggests similar behaviour during translocation, which could be caused by the physico-chemical similarities between the elements. All four elements are known to be trivalent during translocation which might imply the complexation by the same chelating agent^66^. In this case, citrate is a likely candidate, since Fe and Al are generally complexed by citrate during translocation^67,68^. Likewise, organic acids have been found to be important for the long-distance transport of REEs^48,69,70^. For instance, Liu et al.^65^, who analysed organic acid secretion by *Phytolacca americana* grown in a nutrient solution doped with Y, suggest that citrate complexes are the chemical form for Y translocation.

**Figure 7:**
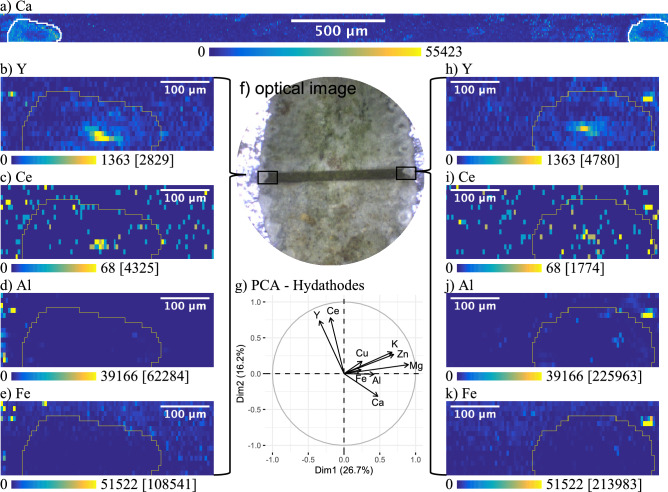
Scaled LA-ICP-MS elemental maps (arbitrary unit) for Ca, Y, Ce, Al and Fe of a leaf transect covering two hydathodes of commercial *Saxifraga paniculata* grown on Y doped soil and the PCA loadings plot for both hydathodes; (**f**) is an optical image of the leaf and the ablated region; (**a**) shows the whole elemental map of Ca with the hydathodes encircled in white (no adjustment of contrast); (**b–e**) are elemental maps of Y, Al, Fe and Ce of the left hydathode; (**h–k**) are elemental maps of Y, Al, Fe and Ce of the right hydathode; (**g**) shows the loadings plot of the PCA performed on all pixels within the ROIs illustrated by the yellow outline on each map; Spot-size: 10 µm; Frequency: 10 Hz; Scan-speed: 10 μm/s; Laser power density: 3.74 GW/cm^2^; Fluence: 18.72 J/cm^2^; Contrast: 0.3% of saturated pixels; Maximum pixel values are indicated in brackets.

The fact that Y remained colocalized with the same elements in all three plant parts analysed suggests that there is no change in Y speciation during transport. However, a close look on the elemental maps of the leaves, shows irregularities in the distribution of elements around the hydathodes (Fig. 7). Hydathodes are openings on the leaf surface through which guttation takes place. Guttation again is the regulation of root pressure through the secretion of droplets which is performed by a wide range of plant species and fungi^71^. Guttation fluid contains metabolites, enzymes and hormones and debate is ongoing as to whether this process is part of a metal resistance strategy^72,73^. In some species of the genus *Saxifraga*, the repeated evaporation of the guttation liquid results in the formation of a Ca-rich crust surrounding the hydathodes^74^. Figure 7a shows the elemental map of Ca obtained by LA-ICP-MS from a leaf-transect that covers two hydathodes of commercial *S. paniculata*. The two hydathodes can be identified by marked accumulation of Ca. Interestingly, in the ROI that contains only the two hydathodes, Y and Ce are no longer correlated with Al or Fe (Fig. 7g). In this ROI, Y and Ce are localised in the centre of the hydathodes while Al and Fe are not visible (Fig. 7b,c,h,i). This could mean that Y and Ce are secreted by guttation, whereas Al and Fe enter the leaf cells before being secreted. This separation could be explained by xylem unloading of Fe and Al in the leaves. For Fe this implies a speciation change induced by ferric-chelate-reductase^66,75^. Al on the other hand, might be stored in vacuoles after undergoing a transformation from Al-citrate to Al-oxalate^76,77^. Since the hydathodes are representing the only plant tissue in which REEs are not colocalized with Al and Fe, it would be interesting to conduct further studies to explore the importance of guttation for plant internal handling of REEs and its influence on REE speciation.

## Conclusion

In this study, two populations of *Saxifraga paniculata* (*S. paniculata*) were grown on an yttrium (Y) doped soil to evaluate their accumulation potential and to explore Y uptake by plants.

Wild *S. paniculata* accumulated significantly higher concentrations of Y in its leaves and roots than the commercial plants. However, only a small proportion of Y was translocated to the leaves and the concentrations remained well below the hyperaccumulation threshold. Nevertheless, *S. paniculata* might have a potential for REE accumulation if exposed to a mixture of REEs.

Despite differences in Y concentrations between the two populations, plant internal handling of Y appeared to be the same. µXRF and LA-ICP-MS showed that Y mainly remained in the epidermis region. We concluded that Y might be retained by mucilage or fixed to carboxylic groups in the cell walls of the epidermis.

Regarding the transporter or channel involved in Y uptake by the roots, we consider it unlikely that Y enters through Ca channels, as generally assumed for REEs. Rather, it seems likely that Al transporters are involved in Y uptake since both elements are similarly distributed in the elemental maps of root cross sections. Once inside the root, Y was colocalized with Al, Fe and Ce in plants of both origins. This colocalization persisted in the stem and the leaves which strongly suggests a correlated uptake pathway between these elements. We suspect that a common chelating agent like citrate with an affinity for trivalent cations complexes all four elements during transport.

Focussing on the hydathodes, situated on the leaf surface, revealed that Al and Fe were not secreted by guttation and that Y and Ce formed a hotspot localised in the centre of the hydathode. This suggests a speciation change induced by leaf internal processes leading to the separation of the colocalized group of elements.

Our study elucidates the complexity of elemental interactions that occur during uptake by plants and underlines the importance of studying many different plant species and populations. Nanoscale resolution µXRF revealed fascinating differences in elemental uptake and has a promising potential for the elucidation of plant internal processes.

## Supplementary Information

Supplementary Information.

## Data Availability

The datasets generated during and/or analysed during the current study are available from the corresponding author on reasonable request.
