# Supplementary material for: Multiscale imaging on Saxifraga paniculata provides new insights into yttrium uptake by plants
Source: Sci Rep. 2022 Oct 30;12:18268. doi: 10.1038/s41598-022-23107-x (PMC9618566; doi:10.1038/s41598-022-23107-x)
Supplement: Supplementary file 1 — Supplementary Information. [file 41598_2022_23107_MOESM1_ESM.docx]

Supplementary material - Multiscale imaging on *Saxifraga paniculata* provides new insights into yttrium uptake by plants

Till Fehlauer^1^, Blanche Collin^1^, Bernard Angeletti^1^, Mohammad Mustafa Negahi^1^, Cédric Dentant^2,3^, Perrine Chaurand^1^, Claire Lallemand^1^, Clement Levard^1^, Jérôme Rose^1^

1: Aix Marseille Univ., CNRS, IRD, INRAE, Coll. France, CEREGE, Aix-en-Provence, France

2: Parc national des Écrins, Domaine de Charance, 05000 Gap, France

3: Univ. Grenoble Alpes, CNRS, Sciences Po Grenoble, Pacte, 38000 Grenoble, France

Corresponding author: [fehlauer@cerege.fr](mailto:fehlauer@cerege.fr) (Till Fehlauer); [collin@cerege.fr](mailto:collin@cerege.fr) (Blanche Collin)

**SM Table 1:** Soil parameters of LUFA Spayer standard soil 2.4

| **Standard soil type no.** | **2.4** |
| --- | --- |
| Batch No. (Sp=stored; F= field fresh) | Sp2.4 1919 |
| Sampling date | 06/05/2019 |
| Organic carbon (% C) | 1.83 ± 0.25 |
| Nitrogen (% N) | 0.23 ± 0.02 |
| pH value (0.01 M CaCl**2)** | 7.5 ± 0.1 |
| Cation exchange capacity (meq/100g) | 17.6 ± 1.0 |
| Maximum water holding capacity (g/100g) | 45.6 ± 2.7 |
| Weight per volume (g/1000ml) | 1206 ± 58 |
| Presieving (mm) | 10 |
| Final sieving (mm) | 2 |
| Dry matter (%) | 85.6 |
| Water content (g/100gsoil) | 16.8 |
| Water capacity (%) | 36.8 |
|  |  |
| **Particle size distribution (mm) according to German DIN (%):** | |
| <0.002 | 24,5 ± 1.8 |
| 0.002 - 0.006 | 7.8 ± 0.6 |
| 0.006 - 0.02 | 14.8 ± 1.2 |
| 0.02 - 0.063 | 25.3 ± 2.5 |
| 0.063 - 0.2 | 20.4 ± 1.1 |
| 0.2 - 0.63 | 5.6 ± 1.8 |
| 0.63 - 2.0 | 1.5 ± 0.4 |
| Soil type | sandy loam (sL) |
|  |  |
| **Particle size distribution (mm) according to USDA (%):** | |
| <0.002 | 24.5 ± 1.8 |
| 0.002 - 0.05 | 42.9 ± 1.35 |
| 0.05 - 2.0 | 32.6 ± 2.0 |
| Soil type | loam |

**SM Table 2:** Average detection limits and quantification limits based on handling blank as well as coefficients of variation and recovery for internal (n = 36, runs = 7) and external (n = 19, runs = 6) reference samples in ICP-MS; CV = coefficient of variation; DL = detection limit; QL = quantification limit

|  | Limits (µg/L) | | Internal references | | | | External reference | |
| --- | --- | --- | --- | --- | --- | --- | --- | --- |
|  | **Average** | | **EP-L-3 (Tap water)** | | **ES-H-2 (Ground water)** | | **V464 (Oak leaves)** | |
|  | DL | QL | CV | Recovery | CV | Recovery | CV | Recovery |
| **^24^Mg** | 2.34 | 6.82 | 11% | 101% | 4% | 110% | 6% | 96% |
| **^27^Al** | 4.66 | 14.5 | 7% | 105% | 8% | 115% |  |  |
| **^31^P** | 119 | 380 | 67% | 119% | 23% | 119% | 21% | 102% |
| **^39^K** | 15.7 | 44.8 | 21% | 106% | 9% | 110% | 5% | 91% |
| **^43^Ca** | 38.8 | 118 | 9% | 102% | 8% | 112% | 6% | 84% |
| **^55^Mn** | 0.244 | 0.687 | 8% | 106% | 5% | 109% | 6% | 103% |
| **^56^Fe** | 9.22 | 29.4 | 5% | 104% | 4% | 112% | 8% | 96% |
| **^60^Ni** | 2.42 | 6.61 | 5% | 101% | 5% | 106% |  |  |
| **^65^Cu** | 0.141 | 0.424 | 5% | 104% | 4% | 105% | 11% | 86% |
| **^66^Zn** | 0.697 | 1.99 | 4% | 107% | 4% | 113% | 4% | 76% |
| **^89^Y** | 0.007 | 0.020 |  |  |  |  |  |  |
| **^137^Ba** | 0.065 | 0.200 | 6% | 105% | 5% | 98% |  |  |
| **^140^Ce** | 0.005 | 0.013 |  |  |  |  |  |  |

**SM Table 3:** Average total elemental concentrations (in mg/kg DW) and standard deviations (SD) in the different plant parts (Roots, Stems, Leaves) of *Saxifraga paniculata* from the two different origins (Commercial, Wild) after 4 months of growth in an Yttrium doped soil; NA = not available; n = number of replicates

|  |  |  | **Mg** | **Al** | **P** | **K** | **Ca** | **Mn** | **Fe** | **Cu** | **Zn** | **Y** | **Ce** |
| --- | --- | --- | --- | --- | --- | --- | --- | --- | --- | --- | --- | --- | --- |
| **Commercial** (control n=2; doped n=3) | Soil | **Control** | 7904 | 42000 | 758 | 14033 | 51613 | 687 | 24772 | 16.8 | 70.1 | 14.1 | 28.4 |
|  |  | SD | 24.8 | 742 | 7.06 | 91.6 | 1151 | 3.99 | 282 | 0.396 | 1.36 | 0.428 | 1.31 |
|  |  | **Doped** | 8896 | 45078 | 738 | 13939 | 42867 | 628 | 22008 | 16.5 | 79.3 | 373 | 39.1 |
|  |  | SD | 657 | 3954 | 12 | 1024 | 265 | 11.8 | 263 | 0.276 | 1.37 | 17.3 | 3.49 |
|  | Roots | **Control** | 2220 | 4153 | 1477 | 2547 | 31343 | 124 | 3264 | 24.8 | 59.8 | 18.3 | 7.80 |
|  |  | SD | 525 | 78.0 | 20.1 | 541 | 214 | 75.9 | 148 | 4.52 | 27.7 | 12.9 | 1.70 |
|  |  | **Doped** | 1913 | 3434 | 1201 | 2272 | 18798 | 108 | 2191 | 16.2 | 92.5 | 260 | 5.06 |
|  |  | SD | 649 | 647 | 229 | 745 | 8708 | 58.9 | 638 | 2.74 | 68.8 | 85.6 | 1.26 |
|  | Stem | **Control** | 3373 | 115 | 2383 | 7152 | 36009 | 64.7 | 82.9 | 4.65 | 79.4 | 0.224 | 0.241 |
|  |  | SD | 261 | 22.7 | 260 | 2481 | 7517 | 0.760 | 15.4 | 0.980 | 12.6 | 0.101 | 0.142 |
|  |  | **Doped** | 3851 | 260 | 2024 | 10723 | 30955 | 58.8 | 162 | 3.74 | 53.0 | 4.82 | 0.285 |
|  |  | SD | 1186 | 119 | 486 | 7990 | 12877 | 24.3 | 84.9 | 0.906 | 42.0 | 3.95 | 0.148 |
|  | Leaves | **Control** | 5963 | 105 | 1849 | 14131 | 120067 | 90.2 | 89.6 | 3.05 | 13.9 | 0.244 | 0.182 |
|  |  | SD | 1289 | 6.91 | 47.9 | 267 | 9405 | 50.2 | 4.13 | 0.490 | 1.73 | 0.073 | 0.027 |
|  |  | **Doped** | 6989 | 242 | 1363 | 10807 | 76171 | 110 | 154 | 3.16 | 11.4 | 10.2 | 0.378 |
|  |  | SD | 664 | 152 | 199 | 6726 | 12760 | 37.0 | 98 | 0.606 | 1.91 | 7.79 | 0.246 |
| **Wild** (control n=4; doped n=6) | Soil | **Control** | 6089 | 20203 | 752 | NA | 43819 | 626 | 20602 | 16.7 | 70.1 | 7.65 | 16.1 |
|  |  | SD | 176 | 944 | 19.3 | NA | 2016 | 26.0 | 441 | 0.465 | 1.55 | 0.621 | 0.968 |
|  |  | **Doped** | 5875 | 19718 | 778 | NA | 46644 | 706 | 19885 | 16.4 | 68.2 | 216 | 15.2 |
|  |  | SD | 187 | 1183 | 36.9 | NA | 1092 | 280 | 931 | 0.803 | 1.47 | 21.8 | 1.15 |
|  | Roots | **Control** | 3230 | 12240 | 965 | NA | 28590 | 1185 | 9334 | 21.2 | 484 | 8.62 | 17.2 |
|  |  | SD | 1180 | 5529 | 83.3 | NA | 3920 | 438 | 3434 | 3.69 | 234 | 1.99 | 7.51 |
|  |  | **Doped** | 2830 | 10961 | 1073 | NA | 25197 | 1002 | 8694 | 22.5 | 451 | 487 | 16.4 |
|  |  | SD | 553 | 3377 | 147 | NA | 4060 | 390 | 2999 | 4.99 | 97.7 | 153 | 4.74 |
|  | Stem | **Control** | 2529 | 815 | 1814 | NA | 35545 | 662 | 776 | 4.05 | 372 | 0.650 | 1.08 |
|  |  | SD | 625 | 116 | 160 | NA | 14926 | 361 | 200 | 0.960 | 253 | 0.091 | 0.205 |
|  |  | **Doped** | 2414 | 558 | 1479 | NA | 37043 | 583 | 576 | 4.16 | 293 | 14.3 | 0.986 |
|  |  | SD | 506 | 385 | 343 | NA | 11959 | 278 | 578 | 1.45 | 148 | 10.0 | 0.712 |
|  | Leaves | **Control** | 5762 | 223 | 872 | NA | 96426 | 472 | 78.4 | 2.59 | 58.0 | 0.824 | 0.897 |
|  |  | SD | 871 | 99 | 133 | NA | 14817 | 392 | 23.4 | 0.578 | 40.7 | 0.433 | 0.412 |
|  |  | **Doped** | 5866 | 183 | 752 | NA | 97120 | 299 | 52.9 | 2.55 | 29.4 | 57.7 | 1.25 |
|  |  | SD | 926 | 60 | 100 | NA | 11445 | 115 | 29.6 | 0.424 | 8.74 | 18.4 | 0.393 |

Note for SM Table 3:

For some chemical elements the total concentrations were strongly impacted by the plant's origin. This difference is due to the fact that the wild plants were collected at the Jas Roux site which has geological background particularly rich in multiple sulfosalt related elements. Therefore, wild *S. paniculata* had accumulated these elements before being transplanted into the Y doped soil. However, this was not the case for Y since its concentration in the soil of Jas Roux is relatively low (4.22±1.04 mg/kg) compared to usual topsoil concentrations (7-60 mg/kg).

**SM Table 4:** Elemental correlations between Yttrium and the other elements isolated from the XRF spectrum; The analysis was performed with the coloc2 plugin in the open source software Fiji^37^

|  |  | **Pearson's R value** | **Li's ICQ value** | **Spearman's rank correlation value** | **Costes**  **P-value** |
| --- | --- | --- | --- | --- | --- |
| **Y** | As | 0.020 | 0.087 | 0.026 | 0.420 |
| **Y** | Br | 0.020 | 0.065 | 0.008 | 0.640 |
| **Y** | Ca | 0.090 | 0.042 | 0.096 | 0.940 |
| **Y** | Cl | 0.000 | 0.084 | 0.003 | 0.480 |
| **Y** | Cr | 0.030 | 0.077 | 0.017 | 0.690 |
| **Y** | Cu | 0.090 | 0.041 | 0.049 | 0.930 |
| **Y** | Fe | 0.180 | 0.123 | 0.193 | 1.000 |
| **Y** | Ga | 0.020 | 0.082 | 0.019 | 0.290 |
| **Y** | K | 0.060 | 0.050 | 0.079 | 0.850 |
| **Y** | Mn | 0.060 | 0.061 | 0.046 | 0.890 |
| **Y** | Ni | 0.030 | 0.057 | 0.025 | 0.490 |
| **Y** | Rb | 0.070 | 0.015 | 0.091 | 0.990 |
| **Y** | Sr | 0.080 | 0.058 | 0.061 | 0.960 |
| **Y** | Ti | 0.020 | 0.087 | 0.036 | 0.530 |
| **Y** | Zn | 0.090 | 0.065 | 0.102 | 0.850 |

**SM Figure 1:** Phytotron conditions during the growing period

**SM Figure 2:** Correlation between total concentrations of different chemical elements in the roots of *Saxifraga paniculata* of wild and commercial origin (n=9) illustrated by a correlation plot after Spearman's rank coefficient with significant values indicated by asterisks (significance levels = 0.5, 0.01, 0.001; p.adjust = Benjamini & Hochberg)

**SM Figure 3:** Fit (red) of µXRF data (black) of the flyscan on root cross section of wild *S. paniculata* with the peaks for the K-edges of Ca, Mn, Fe, Zn and Y used for the extraction of elemental maps; Exposition-time: 40 ms; Gap-size: 100 µm*60 µm; The peaks used for the fit were Fe (K, L), Ca (K), Y (K, L), Ni (K, L), Cu (K, L), Zn (K, L), K (K), Mn (K, L), As (K, L), Sc (K), Ti (K), Cd (L), Ba (L), Ar (K, L), Kr (K, L), Cr (K, L), Sr (K, L), Rb (K, L), Zr (K, L), Br (K, L), Se (K, L), Cl (K), Ga (K, L), V (K), Co (K, L), S (K), Ge (K, L), Sn (L), Sb (L), Al (K)

| 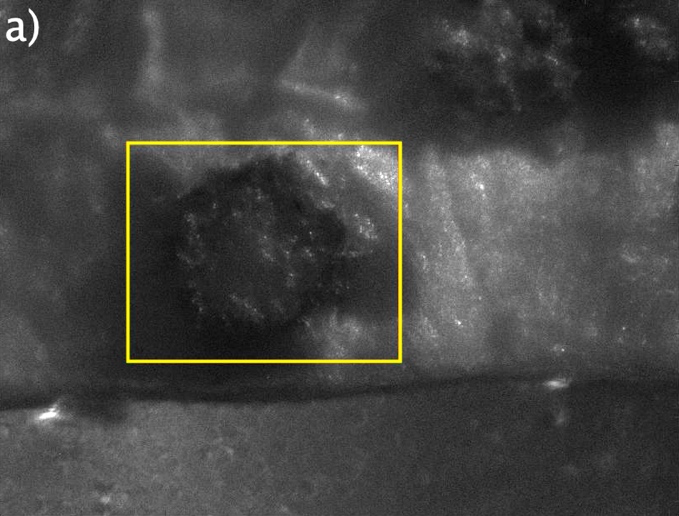 | 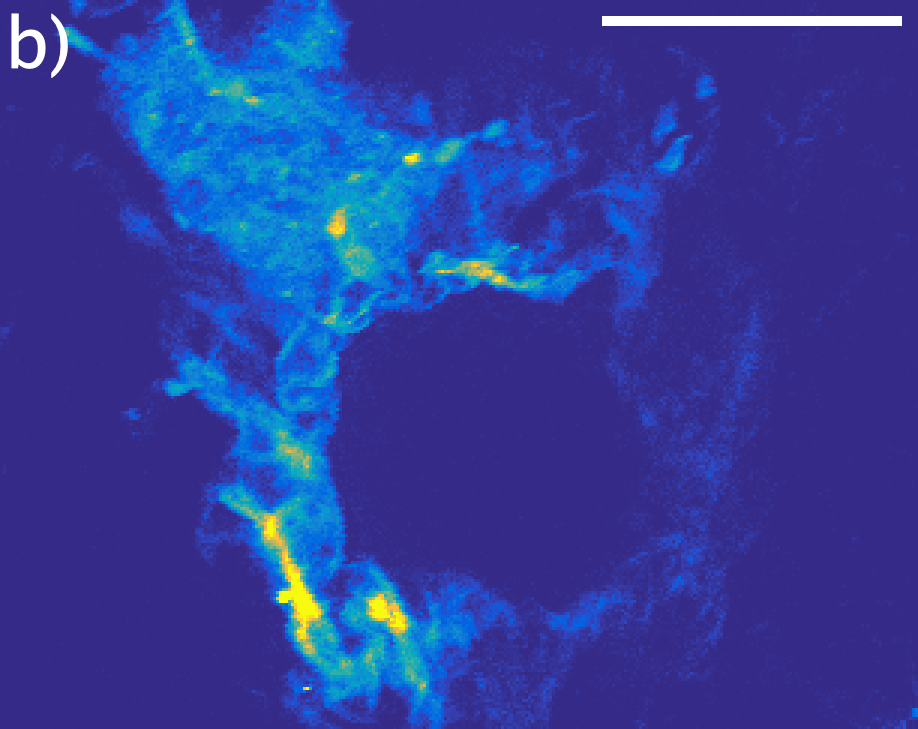 |
| --- | --- |
|  | 0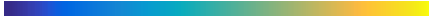 91 [336] |
| 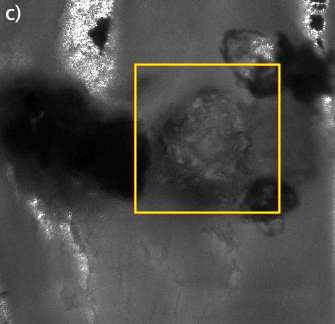 | 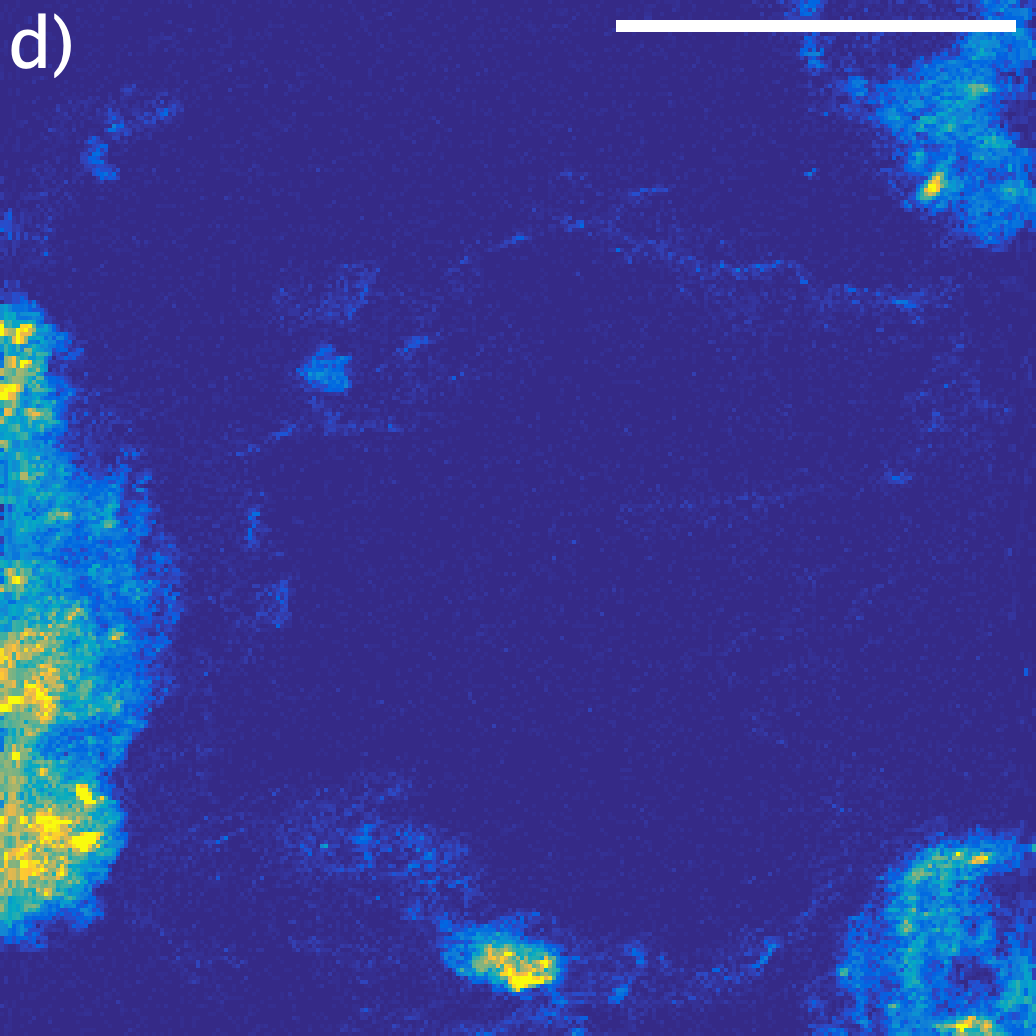 |
|  | 0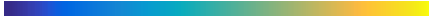 245 [476] |

**SM Figure 4:** Optical images (a, c) with the selected zones and corresponding elemental maps of Y (b, c) obtained with µXRF (arbitrary unit); The maps show two 100 µm thick root cross sections of commercial *Saxifraga paniculata;* The pixel size was 1µm and the exposition-time was 40 ms per pixel; Colormaps are adjusted to 0.3% of saturated pixels and the maximum pixel values are indicated in brackets

**SM Figure 5:** Correlation plots showing Spearman's rank coefficients with significant values indicated by asterisks (significance levels = 0.5, 0.01, 0.001; p.adjust = Benjamini & Hochberg) between the different chemical elements in the different plant parts and adhering soil of commercial *Saxifraga paniculata* (*S. paniculata*)

| 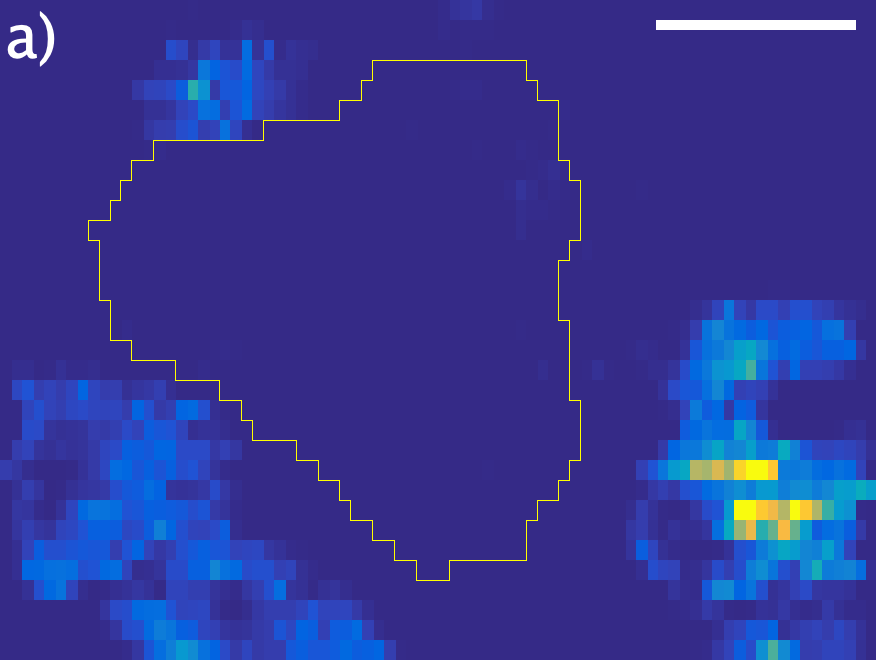 |  | 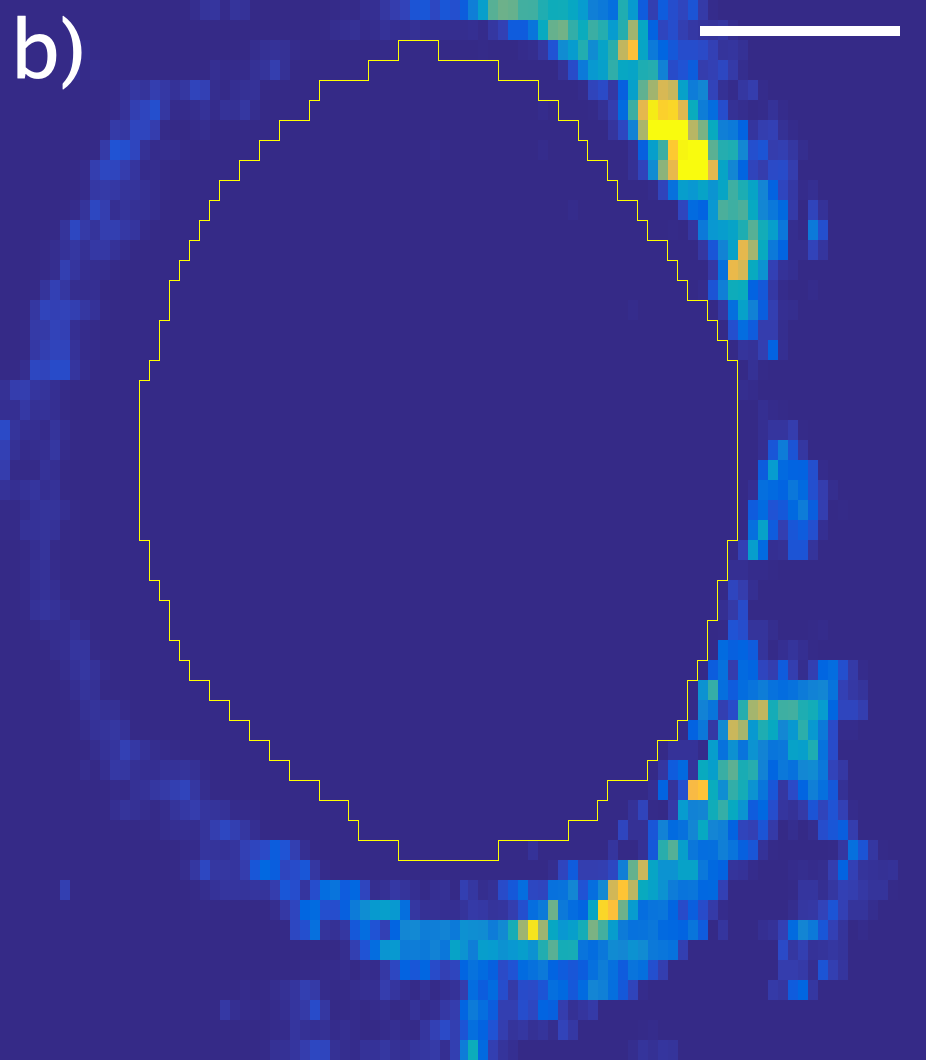 |
| --- | --- | --- |
| 0  400252 [514897] |  | 0  617184 [724766] |
| 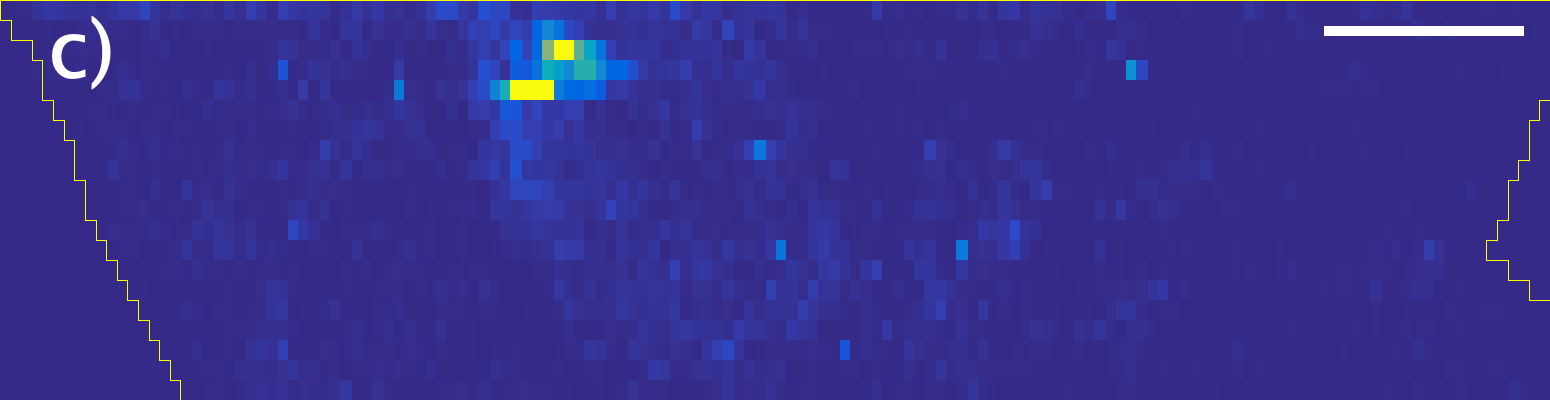 |  | 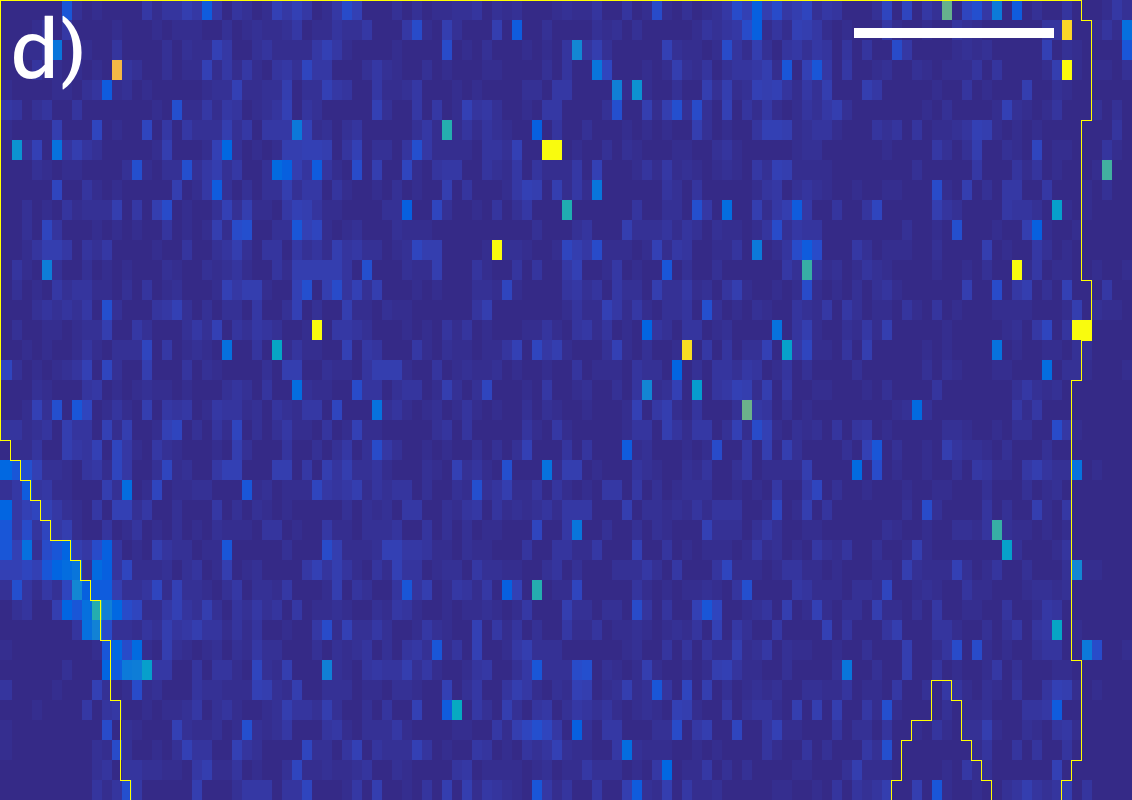 |
| 0  7074 [14724] |  |  |
|  |  | 0  2492 [11601] |
| 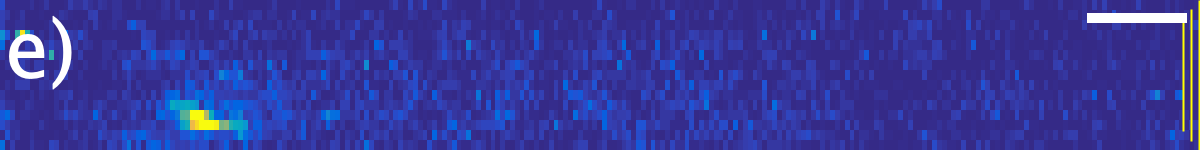 |  | 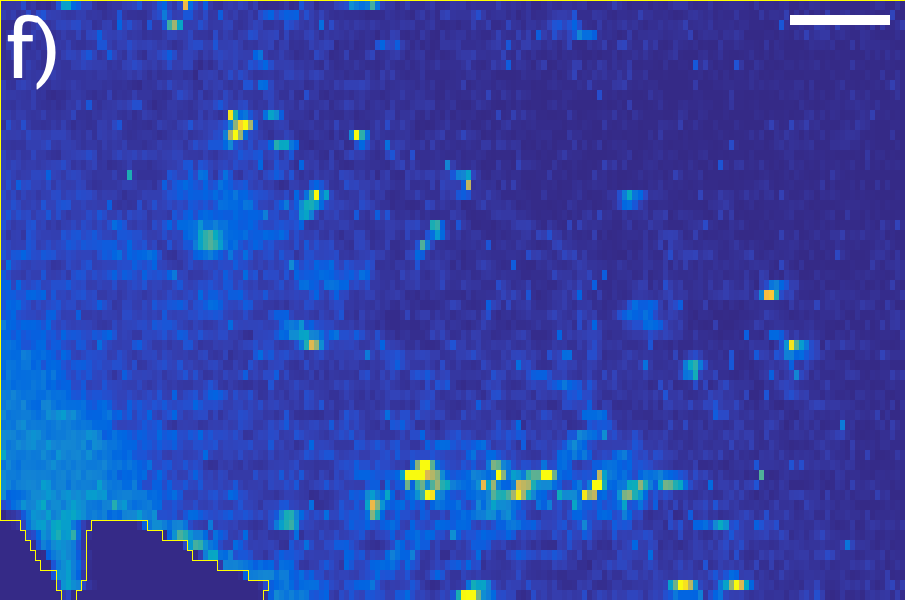 |
|  |  |  |
| 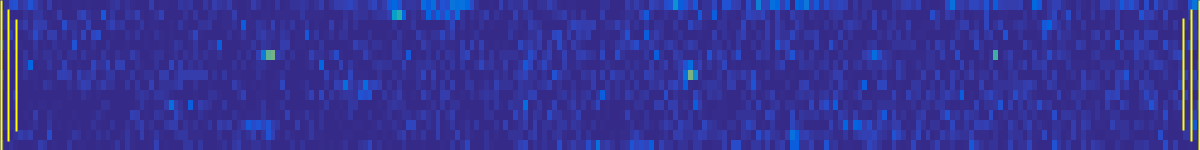 |  |  |
|  |  |  |
| 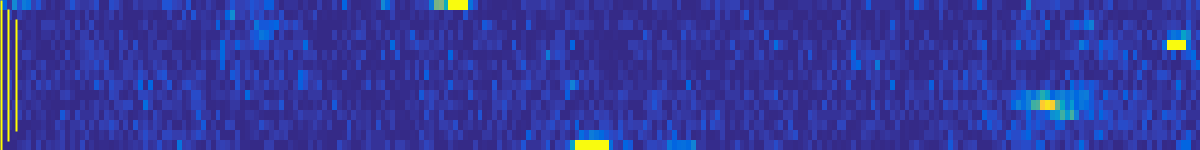 |  |  |
| 0  1363 [58164] |  | 0  251735 [435433] |

**SM Figure 6:** Elemental maps of Y (arbitrary unit) created by LA-ICP-MS for root (a, b), stem(c, d) and leaf (e, f) samples of commercial (a, c, d) and wild (b, d, e) *Saxifraga paniculata* (*S. paniculata*); Colormaps are adjusted to 0.3% of saturated pixels and maximum pixel values are indicated in brackets; The regions of interest selected for the PCAs are outlined in yellow; The map of the leaf transect e) was cut into three parts indicated by the yellow stripes at the border; Scale bars: 100µm

| 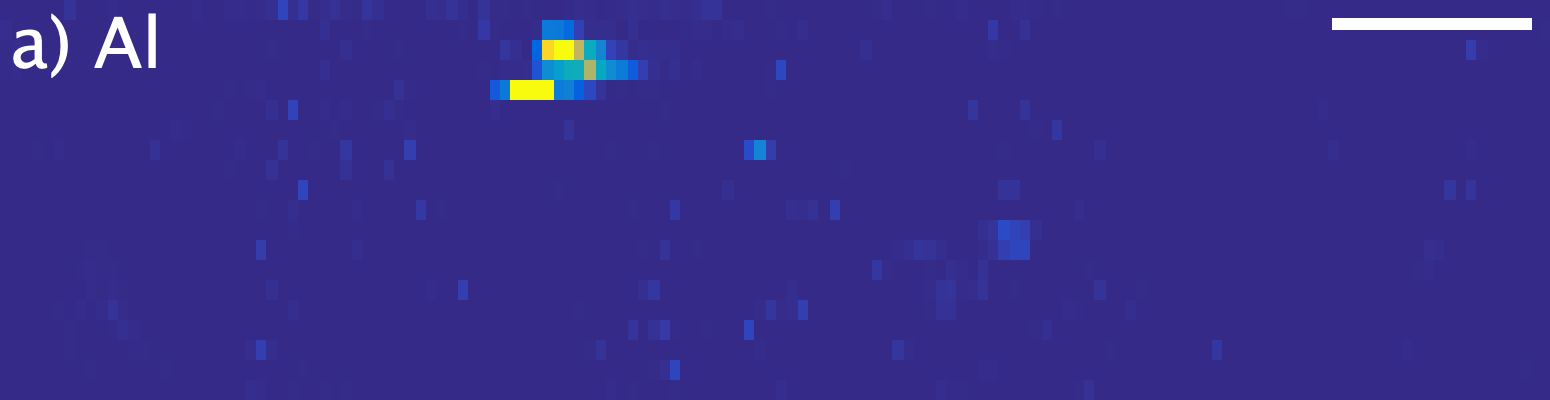 |
| --- |
| 0  400492 [4271917]  arbitrary unit |
| 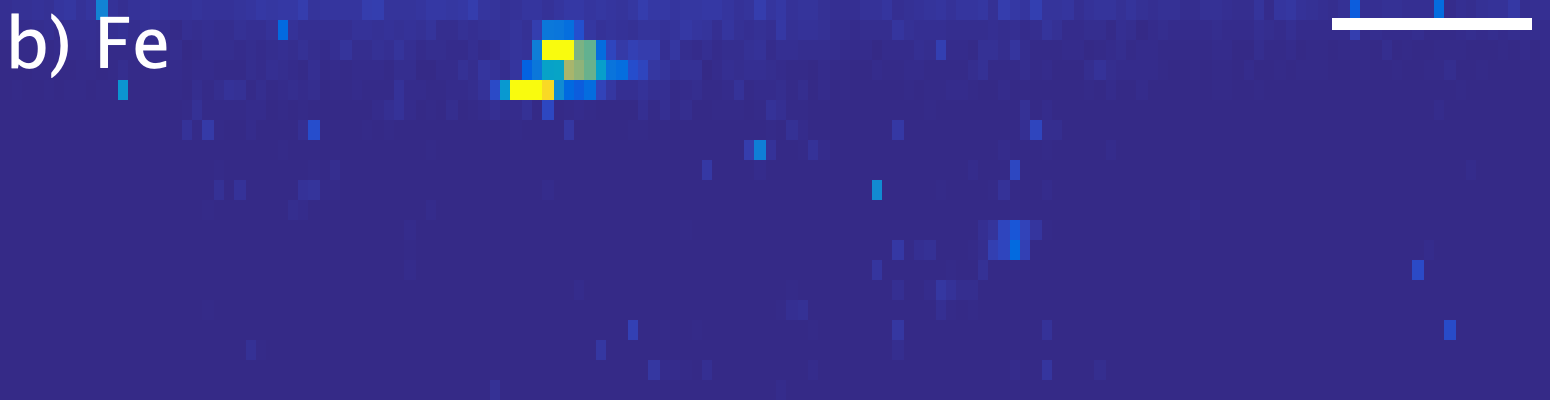 |
| 0  411177 [4210450]  arbitrary unit |
| 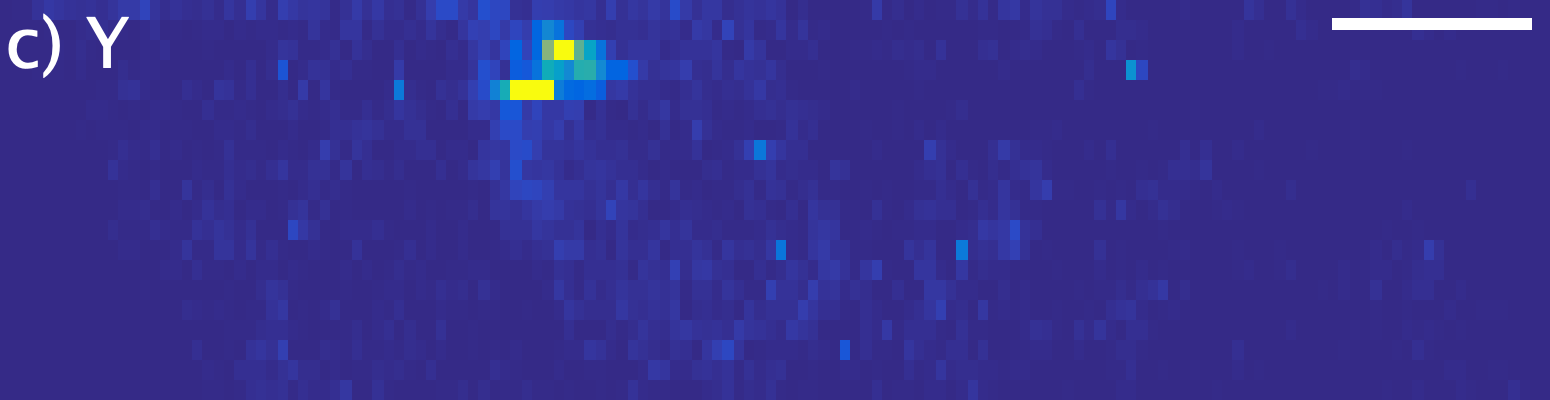 |
| 0  7074 [14724]  arbitrary unit |
| 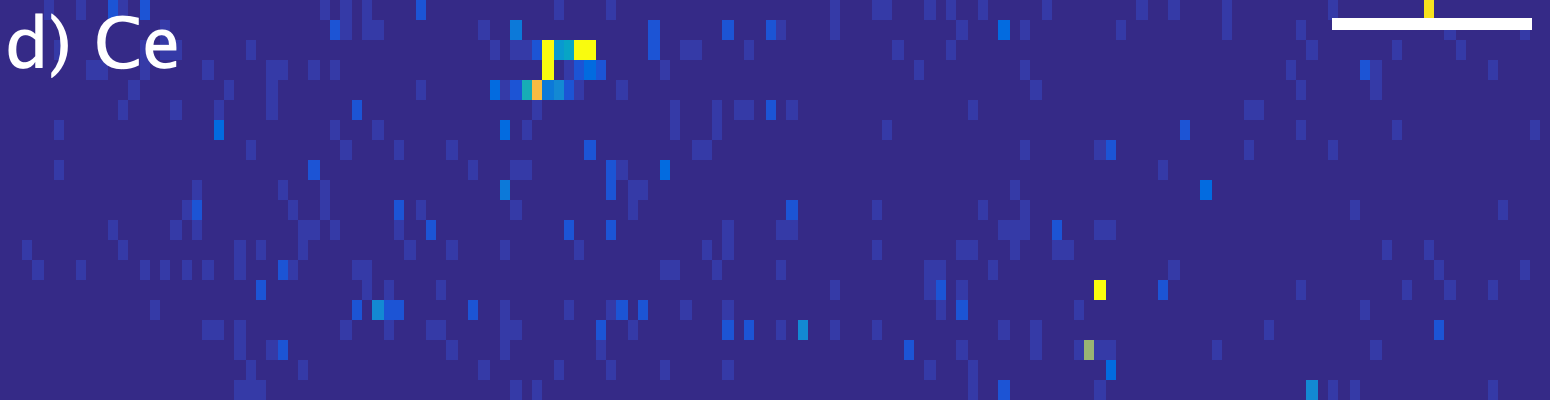 |
| 0  422 [924]  arbitrary unit |

**SM Figure 7**: LA-ICP-MS generated elemental maps of a) Al, b) Fe, c) Y and d) Ce of a stem transect of commercial *Saxifraga paniculata*; The colormaps are adjusted to 0.3% of saturated pixels; The maximum pixel values are indicated in brackets behind the colormap maxima
